# The mystery of the ice cold rose—Microbiome of an Arctic winter frost flower

**DOI:** 10.1002/mbo3.1345

**Published:** 2023-02-04

**Authors:** Stefan Thiele, Anna Vader, Lise Øvreås

**Affiliations:** ^1^ Department of Biological Science University of Bergen Bergen Norway; ^2^ Polar Climate research group Bjerknes Centre for Climate Research Bergen Norway; ^3^ Department of Arctic Biology University Center in Svalbard, UNIS Longyearbyen Norway

**Keywords:** arctic microbes, *Cand*. Nitrosopumilus, microbial ecology, SAR11, sea ice, The Nansen Legacy

## Abstract

Under very cold conditions, delicate ice‐crystal structures called frost flowers emerge on the surface of newly formed sea ice. These understudied, ephemeral structures include saline brine, organic material, inorganic nutrients, and bacterial and archaeal communities in their brine channels. Hitherto, only a few frost flowers have been studied during spring and these have been reported to be dominated by *Rhizobia* or members of the SAR11 clade. Here we report on the microbiome of frost flowers sampled during the winter and polar night in the Barents Sea. There was a distinct difference in community profile between the extracted DNA and RNA, but both were dominated by members of the SAR11 clade (78% relative abundance and 41.5% relative activity). The data further suggested the abundance and activity of *Cand*. Nitrosopumilus, *Nitrospinia*, and *Nitrosomonas*. Combined with the inference of marker genes based on the 16S rRNA gene data, this indicates that sulfur and nitrogen cycling are likely the major metabolism in these ephemeral structures.

## Graphical abstract

Here we report on the microbiome of frost flowers sampled during the winter and polar night in the Barents Sea. There was a distinct difference in community profile between the extracted DNA and RNA, but both were dominated by members of the SAR11 clade (78% relative abundance and 41.5% relative activity). The data further suggested high abundance and activity by *Cand*. Nitrosopumilus, *Nitrospinia*, and *Nitrosomonas*, indicating that sulfur and nitrogen cycling are likely the major metabolism in these ephemeral structures.

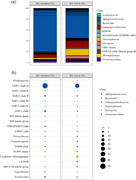

## 1 INTRODUCTION

Frost flowers arise under very cold and calm conditions when water vapor from brine expelled from white or black nilas ice crystallizes on the sea ice surface. As these ephemeral structures grow, they wick up brine, quickly becoming highly saline (up to ~10% salinity). Frost flowers can also contain high concentrations of sulfate (~50 µM), nitrate (~22 mM), dissolved organic carbon, and colored dissolved organic matter (Beine et al., 2012; Douglas et al., 2012). They have been suggested to be the main source of salinity in aerosols, a hotspot for photochemical reactions, and foster ocean—sea ice—atmosphere interactions (Beine et al., 2012; Douglas et al., 2012). Due to the fragile and short‐lived nature of frost flowers, as well as their formation only on thin ice, which is difficult to sample, very few studies have addressed sea ice frost flowers before. Most of which are from coastal or land‐fast ice, and never from open ocean sea ice. Bacterial and archaeal numbers are 3–6 times elevated in frost flowers as compared to the underlying ice as they are wicked into the frost flower during flower growth (Bowman & Deming, 2010; Eronen‐Rasimus et al., 2014). This was shown for frost flowers in Barrow, Alaska, where the cell abundance was 1.28 × 10^5^ mL^−1^ in the frost flowers as compared to 0.28–3.83 × 10^5^ mL^−1^ in the underlying ice. Frost flowers from the central Arctic Ocean harbored even more cells with 3.46 × 10^6^ mL^−1^ (Bowman & Deming, 2010). This abundance is correlated with higher salinity and results in high concentrations of cryoprotectant exopolymers in the frost flowers (Bowman & Deming, 2010). The process of bacterial and archaeal acquisition is selective and leads to communities with a different composition than sea ice or water (Bowman & Deming, 2010; Bowman et al., 2013). In coastal frost flowers from spring in Barrow, Alaska, a dominance of *Rhizobia* (Bowman et al., 2013) or *Propionibacterineae* (Mortazavi et al., 2015) was found. In frost flowers from coastal Greenland in spring, SAR11, *Nitrospina* and *Rhodobacteraceae* were dominant (Barber et al., 2014). Here we report for the first time the bacterial and archaeal community composition from frost flowers sampled on the Northern Barents Sea during the dark polar night, showing community differences in comparison to coastal spring samples.

## 2 MATERIALS AND METHODS

The sample was collected during the RV “Kronprins Haakon” cruise 2019711 on December 9, 2019, at station NLEG09 (79.2434 N, 34.342 E), from a cage, lowered to just above the ice surface, frost flowers were scooped into a sterilized bucket from two areas of about 5 m² size using a sterilized spade. The samples were thawed at 4°C, and the resulting 4.2 L were filtered on a 0.22 µm Sterivex filter, and stored at −80°C. 1.8 mL glutaraldehyde fixed thawed frost flowers were used to determine the cell abundance using a FACS Calibur (Becton Dickinson) flow cytometer according to Marie and coworkers (Brussaard, 2004; Marie et al., 1999). The DNA and RNA were extracted using the Qiagen AllPrep DNA/RNA Mini Kit according to the manual. The extracted RNA was treated with the DNA‐free DNA Removal kit (Life Technologies) and reverse‐transcribed using the SuperScript III First‐Strand Synthesis kit (Life Technologies) according to the manual. Sequencing libraries targeting the V4 region were prepared using the primers 515F—5′‐GTGYCAGCMGCCGCGGTAA‐3′ and 806 R—5′‐ GGACTACNVGGGTWTCTAAT‐3′ (Apprill et al., 2015; Parada et al., 2016) and sent to the Norwegian Sequencing Centre for sequencing on a MiSeq platform with paired‐end reads of 2 × 250 bp lengths. The raw sequences were deposited at the European Nucleotide Archive as project PRJEB57286. Amplicon sequence variants (ASVs) were generated using DADA2 (Callahan et al., 2016), chimeras were removed, and the taxonomy was assigned to the 64412 16S rRNA gene (DNA) and 91859 16S rRNA sequences (RNA) based on the Silva SSU Ref NR v138 (Quast et al., 2013). Thereafter, ASVs of mitochondria, chloroplast, or eukaryote origin were removed. The genetic potential was inferred using PiCRUST2 (Douglas et al., 2020) to detect the occurrence of marker genes, thereby indicating specific metabolisms. The analyses were done using R 4.2.1 in R Studio 2022.07.02 with the packages “vegan 2.6‐4,” “tidyverse 1.3.1,” “phyloseq 1.40.0,” “ggplot2 3.3.6,” and “ape 5.6‐2” (Mazerolle, 2020; McMurdie & Holmes, 2013; Oksanen et al., 2022; Paradis & Schliep, 2019; R core team, 2021; Wickham et al., 2019).

## 3 RESULTS

**Figure 1:**
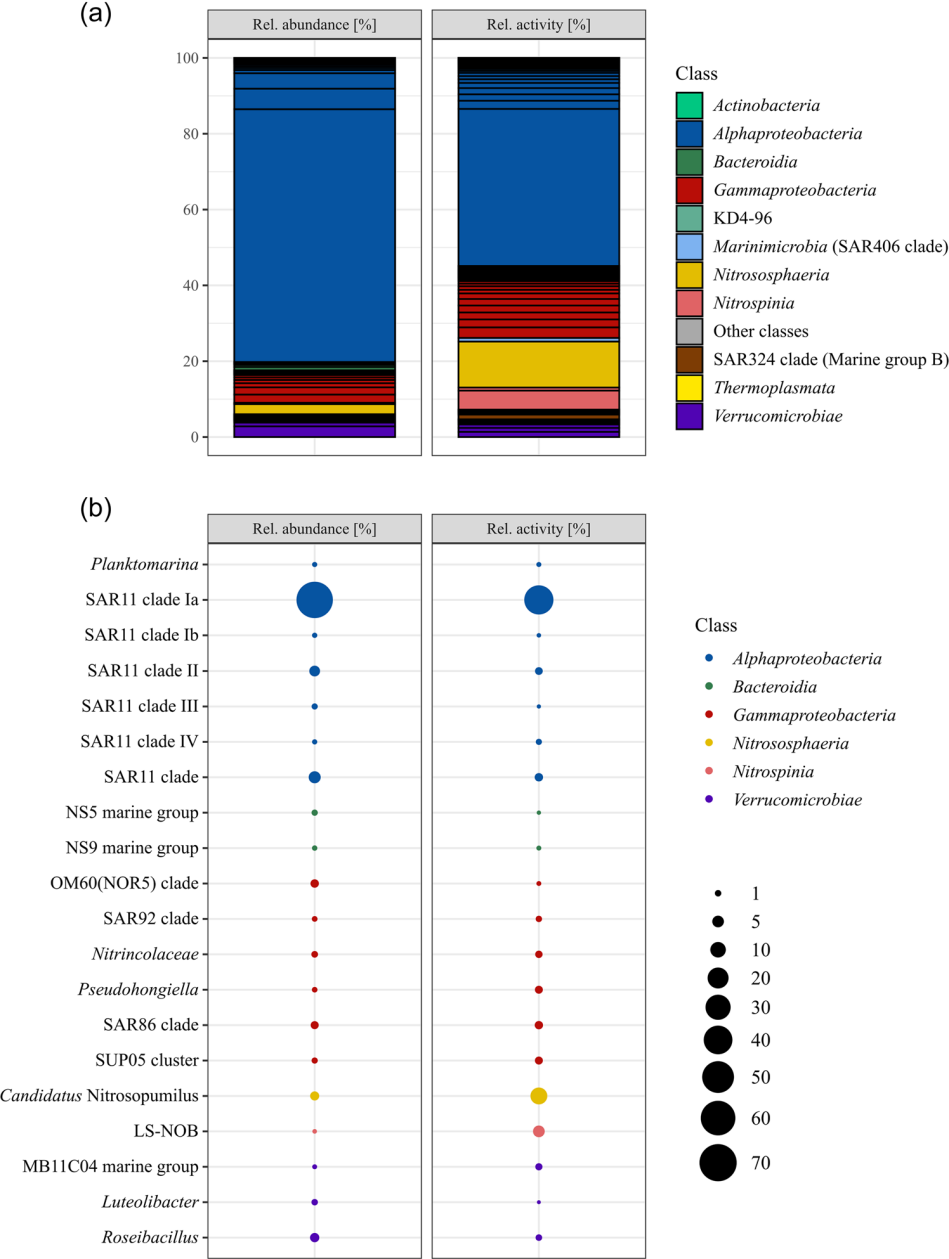
(a) Relative abundance and relative activity of the total bacterial and archaeal community on class level (<0.1% merged into “Other classes”), the black lines mark genera within the class. (b) Relative abundance and relative activity of the 20 most abundant genera, encompassing 94% of DNA sequences and 76% of RNA sequences in the sample.

Serendipitously, a frost flower sample was collected from black nilas ice within the pack ice in the Northern Barents Sea during the polar night. The cell abundance within the frost flower was 2.23 × 10^5^ mL^−1^. The bacterial and archaeal community structure showed a clear dominance of members of the SAR11 clade with 78% relative abundance (defined as the relative abundance of 16 S rRNA gene (DNA) reads), followed by *Roseibacillus* (2.8%), *Candidatus* Nitrosopumilus (2.7%), members of the OM60/NOR5 clade (2.2%), and members of the SAR86 clade (1.9%; Figure 1). All other genera had ≤1% relative abundance. Members of SAR11 subclade Ia constituted 66.7% of the total community and were dominated by a single ASV (57.7%; Figure 1). The relative activity, defined as the relative abundance of 16 S rRNA sequences, was constituted mostly of members of the SAR11 clade Ia with 41.5% dominated by the same ASV with 35.4%. This was followed by *Cand*. Nitrosopumilus (12.1%), LS‐NOB of the family *Nitrospina* (5.0%), and *Nitrosomonas* (2.8%) (Figure 1). *Cand*. Nitrosopumilus and LS‐NOB had much higher abundances in RNA than in DNA reads, while the opposite trend was seen for most members of SAR11 (Figure 1). PiCRUST2 analyses resulted in the inference of *aprA* genes, which represents marker genes for dissimilatory sulfur oxidation or reduction, highest in members of the SAR11 clade (0.18% total inferred gene abundance). In addition, the marker gene for nitrification, *amoA*, was inferred for *Cand*. Nitrosopumilus (0.003% and 0.002%) and *Nitrosomonas* (both 0.001%), while only *nirS/nirK* was inferred for all *Nitrospina* (0.002%). Other marker genes were not inferred or were in very low abundance. The carbohydrate‐active enzyme (CAZyme) classes glycosyl hydrolases (GH) and glycosyl transferases (GT) were inferred for nearly all ASVs, but specifically for *Alpha*‐, *Gammaproteobacteria*, *Bacteroidia*, and *Verrucomicrobiae*.

## 4 DISCUSSION

The bacterial and archaeal community of the frost flowers from the Barents Sea, sampled during the dark polar night, differs substantially from the communities reported previously. The cell abundance in the surface waters (10 m depth) sampled at one of the closest by stations (~50 km distance), was relatively similar to the cell abundance in the frost flower with 2.31 × 10^5^ mL^−1^, while at the other close by station, it was lower with 1.48 × 10^5^ mL^−1^ (Thiele, pers. communication). This is much lower than the numbers reported from summer samples by Bowman and Deming (2010). The difference in the season might lead to differences in surface water cell abundance and also the growth behavior of frost flowers might explain these differences. Another factor could be the age of the frost flowers, with fewer bacteria in young frost flowers. Even though a high relative abundance of members of the SAR11 clade was shown before (Barber et al., 2014), the relative abundance was three times higher in the frost flower community analyzed here. In comparison, the abundance of members of the SAR11 clade at the closest stations was lower (37.8 ± 10.6% relative abundance), although the relative abundance of *Cand*. Nitrosopumilus was higher (11.2 ± 8.1%; Thiele pers. communication). The relative activity of all members of the SAR11 clade was lower than the relative abundance. The inference of the *aprA* gene is due to the abundance of members of SAR11 clades, which are suspected to perform sulfur oxidation for energy and detoxification of sulfite (Meyer & Kuever, 2007; Thiele et al., 2017). This would imply high sulfate concentrations in the frost flower (Beine et al., 2012; Douglas et al., 2012). *Cand*. Nitrosopumilus is reported for the first time in frost flowers but has previously been found in Arctic sea ice and seawater during winter (Thiele et al., 2022; Wilson et al., 2017). *Cand*. Nitrosopumilus and *Nitrosomonas* oxidize ammonia to nitrite and *Nitrospina* might be capable of complete nitrification (Könneke et al., 2005; Koops et al., 1991; Lücker et al., 2013), hence the abundance of *amoA* predicted for these genera. Under anaerobic conditions, all of these taxa could rely on denitrification, as indicated by the abundance of *nirS/nirK* genes (Cantera & Stein, 2007). *Nitrospina* has been found in spring frost flowers, while *Cand*. Nitrosopumilus might have been absent at that time due to photoinhibition (Merbt et al., 2012). Taken together our results indicate that nitrogen and sulfur cycling are the predominant metabolisms in polar night frost flowers. Another factor shaping the bacterial and archaeal community might be the cell size and the size of the brine channels which range from µm to cm scale (Krembs et al., 2000). As frost flowers are intricate, the brine channels are very small and might act as a sieve that only allows entry of small cells, such as those of *Cand*. Nitrosopumilus or members of the SAR11 clade are among the smallest cells in marine environments with average cell diameters of 0.12–0.22 µm (Könneke et al., 2005; Rappé et al., 2002). The inference of GH and GT indicates carbon cycling activities by the main marine carbon degraders *Gammaproteobacteria*, *Bacteroidia*, and *Verrucomicrobiae* (Teeling et al., 2012). However, the low abundance of these classes, suggests that it is not organic carbon cycling, but nitrification which is the main metabolism in sea ice frost flowers in the polar night.

## AUTHOR CONTRIBUTIONS

**Stefan Thiele**: data curation (lead); formal analysis (lead); investigation (lead); methodology (lead); software (lead); visualization (lead); writing – original draft (lead); writing – review and editing (lead). **Anna Vader**: conceptualization (equal); formal analysis (supporting); funding acquisition (supporting); writing – review and editing (supporting). **Lise Øvreås**: conceptualization (lead); formal analysis (supporting); funding acquisition (lead); investigation (lead); writing – review and editing (supporting).

## CONFLICT OF INTEREST STATEMENT

None declared.

## ETHICS STATEMENT

None required.

## Data Availability

The raw sequences are accessible at the European Nucleotide Archive as project PRJEB57286: https://www.ebi.ac.uk/ena/browser/view/PRJEB57286

## References

[mbo31345-bib-0001] Apprill, A. , McNally, S. , Parsons, R. , & Weber, L. (2015). Minor revision to V4 region SSU rRNA 806R gene primer greatly increases detection of SAR11 bacterioplankton. Aquatic Microbial Ecology, 75, 129–137.

[mbo31345-bib-0002] Barber, D. G. , Ehn, J. K. , Pućko, M. , Rysgaard, S. , Deming, J. W. , Bowman, J. S. , Papakyriakou, T. , Galley, R. J. , & Søgaard, D. H. (2014). Frost flowers on young Arctic sea ice: The climatic, chemical, and microbial significance of an emerging ice type. Journal of Geophysical Research: Atmospheres, 119, 11593–11612.

[mbo31345-bib-0003] Beine, H. , Anastasio, C. , Domine, F. , Douglas, T. , Barret, M. , France, J. , King, M. , Hall, S. , & Ullmann, K. (2012). Soluble chromophores in marine snow, seawater, sea ice and frost flowers near Barrow, Alaska. Journal of Geophysical Research: Atmospheres, 117, D00R15.

[mbo31345-bib-0004] Bowman, J. S. , & Deming, J. W. (2010). Elevated bacterial abundance and exopolymers in saline frost flowers and implications for atmospheric chemistry and microbial dispersal. Geophysical Research Letters, 37, L13501.

[mbo31345-bib-0005] Bowman, J. S. , Larose, C. , Vogel, T. M. , & Deming, J. W. (2013). Selective occurrence of Rhizobiales in frost flowers on the surface of young sea ice near Barrow, Alaska and distribution in the polar marine rare biosphere. Environmental Microbiology Reports, 5, 575–582. 23864572 10.1111/1758-2229.12047

[mbo31345-bib-0041] Brussaard, C. P. D. (2004). Optimization of procedures for counting viruses by flow cytometry. Applied and Environmental Microbiology, 70, 1506–1513. 15006772 10.1128/AEM.70.3.1506-1513.2004 PMC368280

[mbo31345-bib-0006] Callahan, B. J. , McMurdie, P. J. , Rosen, M. J. , Han, A. W. , Johnson, A. J. A. , & Holmes, S. P. (2016). DADA2: High‐resolution sample inference from Illumina amplicon data. Nature Methods, 13, 581–583. 27214047 10.1038/nmeth.3869 PMC4927377

[mbo31345-bib-0007] Cantera, J. J. L. , & Stein, L. Y. (2007). Molecular diversity of nitrite reductase genes (nirK) in nitrifying bacteria. Environmental Microbiology, 9, 765–776. 17298375 10.1111/j.1462-2920.2006.01198.x

[mbo31345-bib-0008] Douglas, G. M. , Maffei, V. J. , Zaneveld, J. R. , Yurgel, S. N. , Brown, J. R. , Taylor, C. M. , Huttenhower, C. , & Langille, M. G. I. (2020). PICRUSt2 for prediction of metagenome functions. Nature Biotechnology, 38, 685–688. 10.1038/s41587-020-0548-6 PMC7365738 32483366

[mbo31345-bib-0009] Douglas, T. A. , Domine, F. , Barret, M. , Anastasio, C. , Beine, H. J. , Bottenheim, J. , Grannas, A. , Houdier, S. , Netcheva, S. , Rowland, G. , Staebler, R. , & Steffen, A. (2012). Frost flowers growing in the Arctic ocean‐atmosphere–sea ice–snow interface: 1. Chemical composition. Journal of Geophysical Research: Atmospheres, 117, D00R09.

[mbo31345-bib-0010] Eronen‐Rasimus, E. , Kaartokallio, H. , Lyra, C. , Autio, R. , Kuosa, H. , Dieckmann, G. S. , & Thomas, D. N. (2014). Bacterial community dynamics and activity in relation to dissolved organic matter availability during sea‐ice formation in a mesocosm experiment. MicrobiologyOpen, 3, 139–156. 24443388 10.1002/mbo3.157 PMC3937737

[mbo31345-bib-0011] Könneke, M. , Bernhard, A. E. , de la Torre, J. R. , Walker, C. B. , Waterbury, J. B. , & Stahl, D. A. (2005). Isolation of an autotrophic ammonia‐oxidizing marine archaeon. Nature, 437, 543–546. 16177789 10.1038/nature03911

[mbo31345-bib-0012] Koops, H. P. , Böttcher, B. , Möller, U. C. , Pommerening‐Röser, A. , & Stehr, G. (1991). Classification of eight new species of ammonia‐oxidizing bacteria: *Nitrosomonas communis* sp. nov., *Nitrosomonas ureae* sp. nov., *Nitrosomonas aestuarii* sp. nov., *Nitrosomonas marina* sp. nov., *Nitrosomonas nitrosa* sp. nov., *Nitrosomonas eutropha* sp. nov., *Nitrosomonas oligotropha* sp. nov., and *Nitrosomonas halophila* sp. nov. Microbiology, 137, 1689–1699.

[mbo31345-bib-0013] Krembs, C. , Gradinger, R. , & Spindler, M. (2000). Implications of brine channel geometry and surface area for the interaction of sympagic organisms in Arctic sea ice. Journal of Experimental Marine Biology and Ecology, 243, 55–80.

[mbo31345-bib-0014] Lücker, S. , Nowka, B. , Rattei, T. , Spieck, E. , & Daims, H. (2013). The genome of *Nitrospina gracilis* illuminates the metabolism and evolution of the major marine nitrite oxidizer. Frontiers in Microbiology, 4, 27. 23439773 10.3389/fmicb.2013.00027 PMC3578206

[mbo31345-bib-0042] Marie, D. , Brussaard, C. P. D. , Thyrhaug, R. , Bratbak, G. & Vaulot, D. (1999). Enumeration of marine viruses in culture and natural samples by flow cytometry. Applied and Environmental Microbiology, 65, 45–52. 9872758 10.1128/aem.65.1.45-52.1999 PMC90981

[mbo31345-bib-0015] Mazerolle, M. J. (2020). AICcmodavg: Model selection and multimodel inference based on (Q)AIC(c).

[mbo31345-bib-0016] McMurdie, P. J. and Holmes, S. (2013). phyloseq: An R package for reproducible interactive analysis and graphics of microbiome census data. PLoS ONE, 8, e61217. 23630581 10.1371/journal.pone.0061217 PMC3632530

[mbo31345-bib-0017] Merbt, S. N. , Stahl, D. A. , Casamayor, E. O. , Martí, E. , Nicol, G. W. , & Prosser, J. I. (2012). Differential photoinhibition of bacterial and archaeal ammonia oxidation. FEMS Microbiology Letters, 327, 41–46. 22093004 10.1111/j.1574-6968.2011.02457.x

[mbo31345-bib-0018] Meyer, B. , & Kuever, J. (2007). Molecular analysis of the diversity of sulfate‐reducing and sulfur‐oxidizing prokaryotes in the environment, using *aprA* as functional marker gene. Applied and Environmental Microbiology, 73, 7664–7679. 17921272 10.1128/AEM.01272-07 PMC2168068

[mbo31345-bib-0019] Mortazavi, R. , Attiya, S. , & Ariya, P. A. (2015). Arctic microbial and next‐generation sequencing approach for bacteria in snow and frost flowers: Selected identification, abundance and freezing nucleation. Atmospheric Chemistry and Physics, 15, 6183–6204.

[mbo31345-bib-0043] Oksanen, J. , Simpson, G. L. , Blanchet, F. G. , Kindt, R. , Legendre, P. , Minchin, P. R. , O'Hara, R. B. , Solymos, P. , Stevens, M. H. H. , Szoecs, E. , Wagner, H. , Barbour, M. , Bedward, M. , Bolker, B. , Borcard, D. , Carvalho, G. , Chirico, M. , De Caceres, M. , Durand, S. , … Weedon, J. (2022). *Vegan: Community ecology package*. R package version 2. 6–4.

[mbo31345-bib-0021] Parada, A. E. , Needham, D. M. , & Fuhrman, J. A. (2016). Every base matters: Assessing small subunit rRNA primers for marine microbiomes with mock communities, time series and global field samples. Environmental Microbiology, 18, 1403–1414. 26271760 10.1111/1462-2920.13023

[mbo31345-bib-0022] Paradis, E. and Schliep, K. (2019). ape 5.0: An environment for modern phylogenetics and evolutionary analyses in R. Bioinformatics, 35, 526–528. 30016406 10.1093/bioinformatics/bty633

[mbo31345-bib-0023] Quast, C. , Pruesse, E. , Yilmaz, P. , Gerken, J. , Schweer, T. , Yarza, P. , Peplies, J. , & Glöckner, F. O. (2012). The SILVA ribosomal RNA gene database project: Improved data processing and web‐based tools. Nucleic Acids Research, 41, D590–D596. 23193283 10.1093/nar/gks1219 PMC3531112

[mbo31345-bib-0024] R core team . (2021). A language and environment for statistical computing. R Foundation for Statistical Computing.

[mbo31345-bib-0025] Rappé, M. S. , Connon, S. A. , Vergin, K. L. , & Giovannoni, S. J. (2002). Cultivation of the ubiquitous SAR11 marine bacterioplankton clade. Nature, 418, 630–633. 12167859 10.1038/nature00917

[mbo31345-bib-0026] Teeling, H. , Fuchs, B. M. , Becher, D. , Klockow, C. , Gardebrecht, A. , Bennke, C. M. , Kassabgy, M. , Huang, S. , Mann, A. J. , Waldmann, J. , Weber, M. , Klindworth, A. , Otto, A. , Lange, J. , Bernhardt, J. , Reinsch, C. , Hecker, M. , Peplies, J. , Bockelmann, F. D. , … Amann, R. (2012). Substrate‐controlled succession of marine bacterioplankton populations induced by a phytoplankton bloom. Science, 336, 608–611. 22556258 10.1126/science.1218344

[mbo31345-bib-0027] Thiele, S. , Richter, M. , Balestra, C. , Glöckner, F. O. , & Casotti, R. (2017). Taxonomic and functional diversity of a coastal planktonic bacterial community in a river‐influenced marine area. Marine Genomics, 32, 61–69. 28063827 10.1016/j.margen.2016.12.003

[mbo31345-bib-0028] Thiele, S. , Storesund, J. E. , Fernández‐Méndez, M. , Assmy, P. , & Øvreås, L. (2022). A winter‐to‐summer transition of bacterial and archaeal communities in Arctic sea ice. Microorganisms, 10, 1618. 36014036 10.3390/microorganisms10081618 PMC9414599

[mbo31345-bib-0029] Wickham, H. , Averick, M. , Bryan, J. , Chang, W. , McGowan, L. , François, R. , Grolemund, G. , Hayes, A. , Henry, L. , Hester, J. , Kuhn, M. , Pedersen, T. , Miller, E. , Bache, S. , Müller, K. , Ooms, J. , Robinson, D. , Seidel, D. , Spinu, V. , … Yutani, H. (2019). Welcome to the Tidyverse. Journal of Open Source Software, 4, 1686.

[mbo31345-bib-0030] Wilson, B. , Müller, O. , Nordmann, E.‐L. , Seuthe, L. , Bratbak, G. , & Øvreås, L. (2017). Changes in marine prokaryote composition with season and depth over an Arctic polar year. Frontiers in Marine Science, 4, 95.

